# Serous retinal detachment after trabeculectomy in angle recession glaucoma

**DOI:** 10.3205/oc000037

**Published:** 2015-12-28

**Authors:** Avik Kumar Roy, Debananda Padhy

**Affiliations:** 1 Glaucoma Service, LV Prasad Eye Institute, Patia, Bhubaneswar, India

**Keywords:** angle recession, serous retinal detachment, complication of trabeculectomy

## Abstract

An 18-year-old male with 360 degree angle recession after blunt trauma in his right eye developed uncontrolled intraocular pressure (IOP) despite four antiglaucoma medications (AGM) with advancing disc damage. He underwent trabeculectomy with intraoperative mitomycin-c (MMC) application. There was an intraoperative vitreous prolapse which was managed accordingly. On post-surgery day 1, he had shallow choroidal detachment superiorly with non-recordable IOP. This was deteriorated 1 week postoperatively as choroidal detachment proceeded to serous retinal detachment. He was started with systemic steroid in addition to topical route. The serous effusions subsided within 2 weeks time. At the last follow up at 3 months, he was enjoying good visual acuity, deep anterior chamber, diffuse bleb, an IOP in low teens off any AGM and attached retina. This case highlights the rare occurrence of serous retinal detachment after surgical management of angle recession glaucoma.

## Introduction

Complications are not uncommon after trabeculectomy as the integrity of the globe is permanently distorted. Hypotony and shallow anterior chamber (AC) are particularly troublesome as they are harbinger of more sinister complications like corneal decompensation, cataract development, ostium closure by formation of peripheral anterior synechia and choroidal hemorrhage [1]. This case depicts the rare occurrence of serous retinal detachment resulting from hypotony following trabeculectomy in a case of angle recession glaucoma.

## Case description

**Figure 1:**
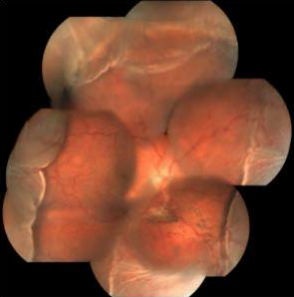
Intraoperative microphotograph showing vitreous prolapse during deep block excision of trabeculectomy

An 18-year-old male was presented to the emergency clinic with a sudden decrease in vision following a blunt trauma with tennis ball 3 days before. His best corrected visual acuity on presentation was hand movement with perception of rays accurate in all quadrants in the right eye. The intraocular pressure (IOP) was digitally high with corneal edema and hyphema in the anterior chamber. He was managed conservatively with topical steroid eye drops, cycloplegics and 2 anti-glaucoma medications namely brimonidine 0.2% and timolol 0.5% twice daily. The hyphema and vitreous hemorrhage settled down in another 3 weeks time when the visual acuity returned to 20/60, angle showed 4-quadrants angle recession, disc showed 0.7 cupping with inferior rim thinning. However the IOP was still 34 mm Hg – hence prostaglandin analogue bimatoprost 0.01% at bed time was added. The IOP did not show substantial improvement over the next 3 weeks, so the patient was advised for filtering surgery under guarded visual prognosis. Intraoperatively there was vitreous prolapse during deep block excision (Figure 1 (Fig. 1)), probably from pre-existing zonular weakness and phacodonesis. This was managed with anterior vitrectomy. 

**Figure 2:**
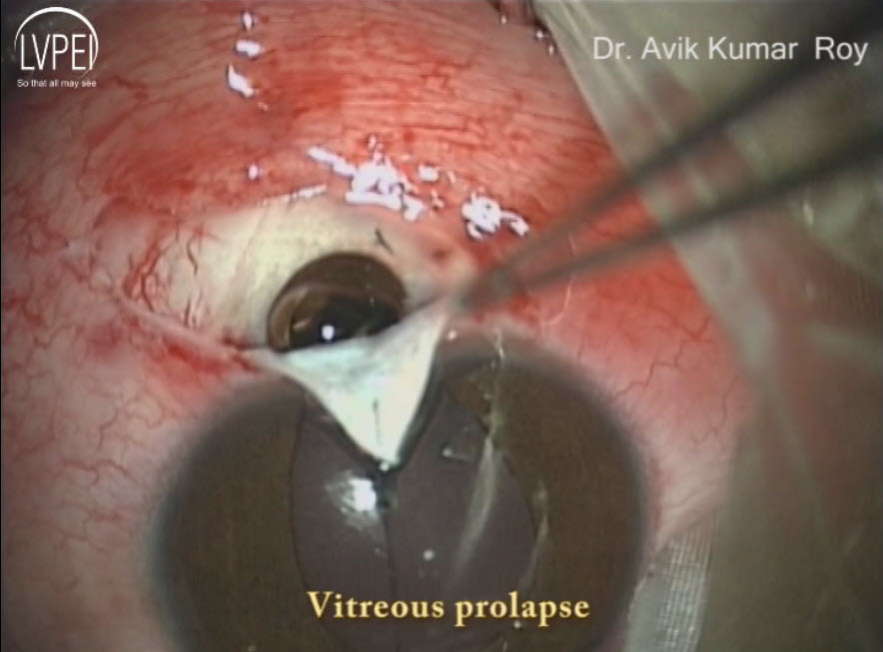
Fundus photograph showing combined choroidal detachment and serous retinal detachment superiorly

**Figure 3:**
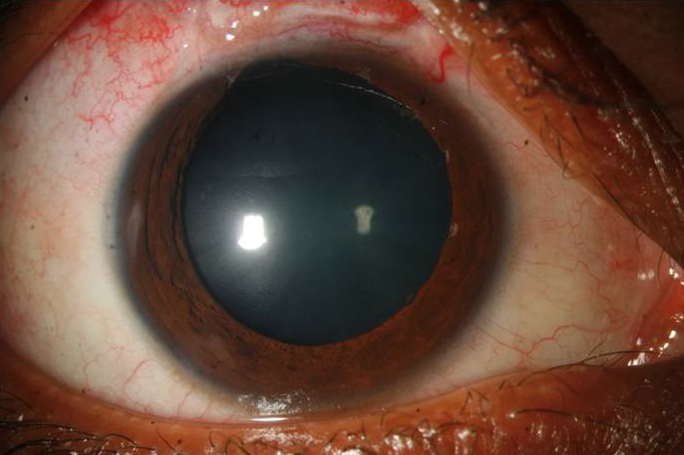
Final slit lamp photograph showing well formed anterior chamber

**Figure 4:**
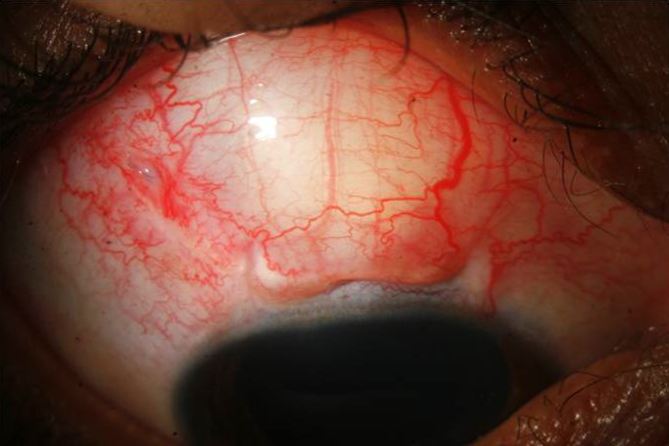
Final slit lamp photograph showing well formed bleb

On postoperative day one, the visual acuity was 20/125 but the IOP was non-recordable. There was serous choroidal detachment which proceeded to frank serous retinal detachment superiorly (Figure 2 (Fig. 2)), with visual acuity dropping to counting finger at 2 meters and IOP being still non-recordable. He was treated with topical prednisolone drop at 2-hourly intervals plus oral prednisolone at 1 mg/kg/day dosage. The effusion settled in 2 weeks time after which the steroids were gradually tapered. Gradually the vision and IOP got improved. At the last follow up visit after 3 months, the best corrected visual acuity and IOP were 20/25 and 12 mm Hg off any medication, respectively (Figure 3 (Fig. 3), Figure 4 (Fig. 4)). 

## Discussion

The occurrence of transient choroidal detachment after trabeculectomy is relatively common. However most of these remain subtle and resolve spontaneously with conservative management [2]. Ocular hypotony alters pressure relationships that normally prevent fluid accumulating in the supra-choroidal space. The risk is increased when there is ocular inflammation – by causing abnormal vascular permeability. The additional component of cyclitis and intraocular penetration of mitomycin-c (MMC) leading to aqueous hyposecretion also contributes to ocular hypotony. Eyes with thickened sclera causing reduced transscleral outflow and eyes with abnormally high hydrostatic pressure as with raised episcleral pressure in Sturge-Weber syndrome (SWS) are more prone to this complication. Interestingly, there are only two cases of serous retinal detachment following trabeculectomy reported up till now – the first one in a 10-year-old girl with SWS [3]. The second case pertains to a 35-year-old gentleman who developed an *isolated* serous retinal detachment two days after glaucoma filtering surgery for his traumatic glaucoma [4]. Management of hypotony is mostly cause-specific. We hypothesized that increased inflammation from intraoperative vitreous loss could have caused the serous effusion in our case. Hence we treated the patient with full steroid therapy and the detachments resolved in 2 weeks time. 

To the best of our knowledge, the complication of combined serous retinal detachment and choroidal detachment after trabeculectomy in angle recession glaucoma has not been previously reported.

## Notes

### Competing interests

The authors declare that they have no competing interests.
